# Dual-modification of biochar *via* Fenton oxidation and *in situ* α-FeOOH synthesis for enhanced Cu(ii) removal: experimental investigation and theoretical calculation analysis

**DOI:** 10.1039/d5ra05887b

**Published:** 2025-11-18

**Authors:** Wei Yang, Ziguo Liu, Qingkun Liu, Qingbin Sun, Han Zheng, Jiancheng Hu

**Affiliations:** a Huangshi HBPU Environmental Protection and Energy Saving Industry Technology Research Institute Co., Ltd Huangshi 435003 China liuziguo@hbpu.edu.cn; b School of Environmental Science and Engineering, Hubei Polytechnic University Huangshi 435003 China; c School of Materials Science and Engineering, Henan Polytechnic University Jiaozuo 454003 China

## Abstract

In the present study, a facile method combining Fenton oxidation and subsequent α-FeOOH deposition for dual-modification of biochar was developed to prepare an efficient adsorbent for removing Cu(ii) from wastewater. Compared to pristine and single-modified biochars, the dual-modified biochar FOBC had a larger adsorption capacity for Cu(ii). Comprehensive investigations encompassing adsorption isotherms, kinetic studies, and thermodynamic analysis showed that the FOBC-based adsorption of Cu(ii) was dominated by an endothermic, monolayer, chemisorption process. Multiple characterization results showed that the primary mechanisms for the adsorption of Cu(ii) were ion exchange and complexation reactions. The α-FeOOH deposited on the FOBC matrix played the most pivotal role during the adsorption of Cu(ii). To explore the adsorption mechanisms at the electron scale, the density functional theory calculations were conducted. The findings suggested that the O^A^-site of α-FeOOH was an extremely robust adsorption site, binding with Cu(ii) to form the bidentate complexes. Moreover, FOBC presented remarkable feasibility, reusability, and structural stability, demonstrating its potential application for treating Cu(ii)-containing wastewater.

## Abstract 2

An innovative composite adsorbent from bamboo biochar was successfully synthesized by dual-modification of Fenton oxidation and surface generation of α-FeOOH and exhibited highly efficient Cu(ii) adsorption.

## Graphical abstract

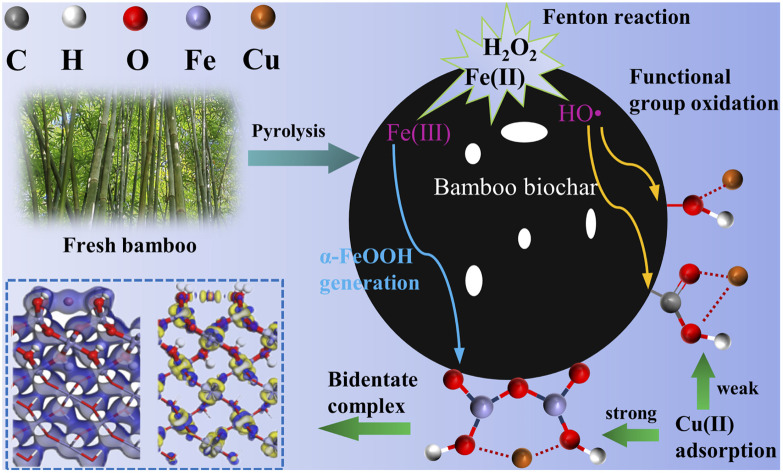

## 1. Introduction

The accelerated emergence of a new energy vehicle industry globally in recent years has rapidly increased demands for heavy metal copper. However, intensive mining and smelting activities of copper mines, as well as the production of new energy vehicle motors, batteries, and circuit boards, can generate large amounts of industrial wastewater containing copper ions [Cu(ii)].^1,2^ This Cu(ii)-containing wastewater can diffuse into habitats through water flow, and can pose serious risks to public health by penetrating the food chain.^3,4^ Previous studies have shown that inordinate intake of Cu(ii) in humans can lead to gastrointestinal irritation, damage to liver, muscular pain, and cancer.^5–7^ Therefore, Cu(ii) should be removed from wastewater before discharging it into larger water bodies.

For the removal of heavy metal ions (HMs) from wastewater, various conventional approaches such as electrochemical treatment, flocculation, chemical precipitation, filtration, and membrane-based treatment have been used.^8,9^ Nevertheless, they are typically costly and labor- and energy-intensive.^10^ Moreover, they can generate large amounts of sludge, which is difficult to be treated (thus resulting in secondary pollution), and therefore, limits their large-scale applications.^11^ In contrast, adsorption has been extensively used in processes involving the removal of heavy metals due to its effectiveness, flexibility, and recyclability.^7,9,12^ However, developing adsorbents with good chemical stability, non-toxicity, low costs, and straightforward synthesis processes remains a challenging task.

As a renewable carbon-enriched material, biochar is produced through high-temperature pyrolysis of biomass.^13^ In recent years, biochar has received substantial interest from researchers due to its adsorption efficiency for HMs.^14–16^ Nevertheless, biochar often possesses relatively low adsorption capacity that stems from its limited quantity of available functional groups and low specific surface area.^10,17^ Therefore, it is imperative to enhance the adsorption capacity of biochar through modification processes. Generally, modification of biochar can be achieved through chemical methods.^18,19^ Indeed, some previous studies have confirmed the enhancement in the adsorption capacity of biochar for HMs through chemical modifications.^9,20^ For instance, the introduction of specific oxygen-containing functional groups on the surface of biochar through oxidation could significantly improve the adsorption capacity of the adsorbent.^10,21^ Shen *et al.*^22^ used perhydrol to oxidize bamboo-derived biochar, resulting in the conversion of C–H/C=C to –COOH on the surface of biochar. Furthermore, compared with unoxidized biochar, they revealed a three-fold enhancement in the adsorption capacity of H_2_O_2_-oxidized biochar for mercury (Hg). Gao *et al.*^23^ modified pine biochar using phosphoric acid to promote the generation of oxygen-containing functional groups (such as –C=O and –COOH) on its surface, and significantly improved the adsorption capacity of the biochar for Cu(ii). Additionally, numerous researchers have used other chemical methods to modify biochar including loading different types of additives (such as metal oxides and hydroxylated iron oxides) on the surfaces of biochar.^16,24^ Some studies and meta-analyses have confirmed that the loading of these additives can significantly enhance the removal efficiency of adsorbents for HMs.^18,19,25^ Li *et al.*^26^ induced the coordination of groups on the surface of biochar with α-FeOOH through iron (Fe) modification, and significantly enhanced the affinity and immobilization capacity of biochar for Hg. Liu *et al.*^27^ synthesized MnO_2_ on the surface of biochar, achieving an adsorption capacity of 187.76 mg g^−1^ for Cu(ii), which was 55.7 times higher than that achieved using the unmodified biochar (3.31 mg g^−1^). Consequently, in order to obtain efficient adsorption of HMs, it is necessary to study how chemical methods can be used to achieve dual-modification of materials. For instance, Chen *et al.*^28^ incorporated α-FeOOH into carboxylated cellulose nanocrystals, which simultaneously resulted in the increase of specific oxygen-based functional groups and loading of active materials on the surface of adsorbent, achieving significant improvements for the adsorption of HMs.

Fenton oxidation refers to the decomposition of H_2_O_2_ through the catalytic activity of Fe(ii) under acidic conditions, which leads to the formation of reactive hydroxyl radicals (HO˙): Fe(ii) + H_2_O_2_ → Fe(iii) + OH^−^ + HO˙.^29^ Hydroxyl radicals are a highly reactive chemical species with a powerful oxidation capability. They can facilitate the oxidation of C–H, C–O–C, and C–C/C=C to –COOH and –OH on the surface of biochar and significantly improve its pore structure.^22^ Furthermore, the generated Fe(iii) can be converted *in situ* to Fe(iii)-derived α-FeOOH by adjusting the pH and temperature of the mixture.^30^ Subsequently, the α-FeOOH undergo complexation with oxygen-containing functional groups, achieving dispersion and fixation of active adsorption materials on the surface of biochar. This dual modification strategy has a synergistic effect, as confirmed by recent research.^28^ Thanks to oxygen-containing functional groups generated by oxidation modification, the aggregation of a-FeOOH on the surface of a-FeOOH-loaded biochar reported in previous study can be avoided.^26,28^ In addition, the problem of the limited number of active adsorption sites on the surface of single oxidation-modified biochar is also simultaneously solved.

In the present study, lignin-rich bamboo is used to synthesize Fenton oxidation modified-biochar loaded with α-FeOOH (FOBC) for Cu(ii) adsorption. The main aim of the work is: (1) to analyze the adsorption performance of prepared adsorbents for Cu(ii); (2) to analyze the mechanisms underlying the FOBC-based adsorption of Cu(ii); and (3) to comprehensively unlock the mechanisms controlling the adsorption reaction at the electron-scale using density functional theory (DFT) calculations. The present work opens up an innovative and simple approach for preparing the dual-modified absorbent, and provides essential theoretical and technological support for the removal of Cu(ii) from wastewater.

## 2. Experimental

### 2.1. Materials

The bamboo specimens utilized in the present study were sourced from Hubei Province, China. Chemicals including CuSO_4_, H_2_SO_4_, NaOH, HCl, FeSO_4_, Fe_2_(SO_4_)_3_, H_2_O_2_ (30 wt%), NaNO_3_, KNO_3_, Al_2_(SO_4_)_3_, CdSO_4_, NiSO_4_, and Cr(NO_3_)_3_, were procured from China National Pharmaceutical Group Chemical Reagents Co., Ltd, and were utilized without any purification. All of these chemicals were of analytical grade. Moreover, Cu(ii) solutions were made by dissolving predetermined quantities of CuSO_4_ in deionized water to achieve specific concentrations. The actual Cu(ii)-containing wastewater used in the present study was collected from a factory in Huangshi, Hubei Province, China.

### 2.2. Preparation of adsorbents

The collected bamboo was first washed and cut into pieces. These pieces were oven-dried at 80 °C for 6 h. The bamboo biochar (BC) was prepared in a tubular furnace by pyrolyzing the dried bamboo fragments at 650 °C for 90 min under the flow of nitrogen gas (100 mL min^−1^). The resulting BC was further processed through mechanical grinding, and sieved through a 0.10 mm mesh.

In order to prepare the Fenton-oxidized biochar, 1 g of produced BC was added to 200 mL of 0.05 M aqueous FeSO_4_ solution. The pH value of the mixed solution was adjusted to 2–3 using a 0.5 M aqueous H_2_SO_4_ solution. Afterward, 50 mL of H_2_O_2_ (30 wt%) was added to the mixture, which was then stirred at 120 rpm and 25 °C for 90 min in a water bath shaker. Finally, the sample was washed using a 1.0 M aqueous HCl solution until no iron ions eluted. The sample was then dried at 80 °C, resulting in Fenton-oxidized biochar (OBC).

In order to prepare α-FeOOH-loaded biochar, 1 g of BC was added to 200 mL of 0.025 M aqueous Fe_2_(SO_4_)_3_ solution. The pH of the mixture was adjusted to 12 using a 1.0 M aqueous NaOH solution. Next, the mixture was aged in a water bath at 60 °C for 12 h. Finally, the precipitates were washed with deionized water until neutral pH was obtained. The resulting sample was the biochar loaded with α-FeOOH (herein abbreviated as FBC).

In order to prepare the dual-modified biochar, 1 g of BC was added to 200 mL of 0.05 M aqueous FeSO_4_ solution. The pH value of the mixture was adjusted to 2–3 using a 0.5 M aqueous H_2_SO_4_ solution. Afterward, 50 mL of H_2_O_2_ (30 wt%) was added to the mixture, which was stirred at 25 °C and 120 rpm for 90 min in a water bath shaker. Subsequently, the pH value of the mixture was further adjusted to 12 using a 1.0 M aqueous NaOH solution. Next, the mixture was aged in a water bath at 60 °C for 12 h before washing the obtained precipitates with deionized water until reaching a neutral pH value. The resulting precipitates were the dual-modified biochar and abbreviated herein as FOBC.

### 2.3. Characterization

The structural characteristics and elemental compositions were examined using scanning electron microscopy-energy dispersive spectroscopy (SEM-EDS; Regulus 8100, Japan). The mineral compositions were evaluated using X-ray diffraction analysis (XRD; D/max-3B, Japan). Specific surface areas, pore volumes, and pore diameters were determined using nitrogen (N_2_) adsorption–desorption isotherms (Micromeritics ASAP2020, USA). Characterization of surface functional groups was carried out using Fourier transform infrared spectroscopy (FTIR; Nicolet iS50, USA), while X-ray photoelectron spectroscopy (XPS; Axis Supra, Japan) was used to characterize the surface chemical states of samples.

### 2.4. Batch experiments

All batch experiments were performed in a fixed volume (100 mL) of Cu(ii) solutions in 200 mL conical flasks that were agitated continuously at 120 rpm. Following the completion of each experiment, a 2 mL supernatant sample was filtered through a 0.22 µm filter paper. The residual Cu(ii) in filtered solutions was quantified using atomic absorption spectroscopy (AAS; PINAACLED 900, PerkinElmer, USA).

To ensure the reliability of measurements, all adsorption experiments were conducted thrice. Experimental outcomes were presented as mean values accompanied by standard errors. The removal efficiency of Cu(ii) (*R*_e_%) was calculated using eqn (1), while the adsorption capacities (mg g^−1^) at equilibrium (*q*_e_) and at time *t* (*q*_*t*_) were determined using eqn (2) and (3), respectively.1
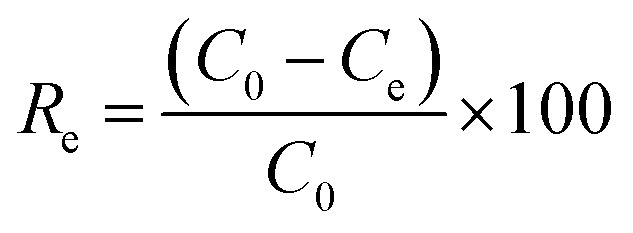
2
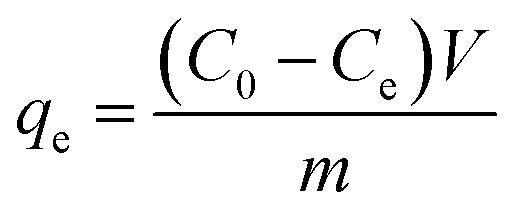
3
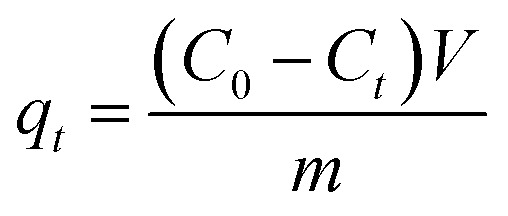
where *C*_0_ (mg L^−1^) denotes the initial concentration of Cu(ii), *C*_e_ and *C*_*t*_ (mg L^−1^) are the concentrations of Cu(ii) at equilibrium and time *t*, *V* (L) denotes the volume of solution, and *m* (g) is the mass of the absorbent.

The equations of the kinetic, isotherm, and thermodynamic models used in the present study are listed in Text S1 of the SI.

### 2.5. DFT calculations

In the present work, DFT calculations were conducted using the CASTEP package (BOVIA Materials Studio). The generalized gradient approximation (GGA) of Perdew–Burke–Ernzerhof (PBE) functional was adopted to describe exchange-functional groups, while the D3 correction approach (DFT-D3) was employed to explore the effects of van der Waals (vdW) interactions. The forces applied on each ion were minimized using the LBFGS algorithm until achieving a value lower than 0.03 eV Å^−1^ to obtain optimized structures. The energy cutoff was set to 1306.10 eV. The convergence thresholds for energy change, maximum stress, maximum displacement, and self-consistent field were set to 1 × 10^−5^ eV per atom, 0.05 GPa, 0.001 Å, and 1 × 10^−6^ eV per atom, respectively. Furthermore, the Monkhorst–Pack *K*-points for the Brillouin-zone integration, with *K*-points of 1 × 2 × 1, were considered in the calculation process.

The (110) facet was cleaved from the unit cell of α-FeOOH to build the slab model for adsorption.^31,32^ Subsequently, a periodic model consisting of a (1 × 2 × 3) supercell was used as the adsorbent, accompanied by a 15 Å-thick vacuum gap.^32^ Meanwhile, Cu(ii) in its ionic state was considered as the adsorbate. The adsorption energy (*E*_ads_) was determined using eqn (4).^31,33^

4 *E*_ads_ = *E*_α-FeOOH+Cu(II)_ − *E*_α-FeOOH_ − *E*_Cu(II)_

where *E*_α-FeOOH+Cu(II)_ represents the combined energy of the α-FeOOH slab and adsorbed Cu(ii); *E*_α-FeOOH_ represents the energy of α-FeOOH slab; *E*_Cu(II)_ is the energy of free Cu(ii) in the vacuum.

## 3. Results and discussion

### 3.1. Physicochemical characteristics of different adsorbents

**Fig. 1:**
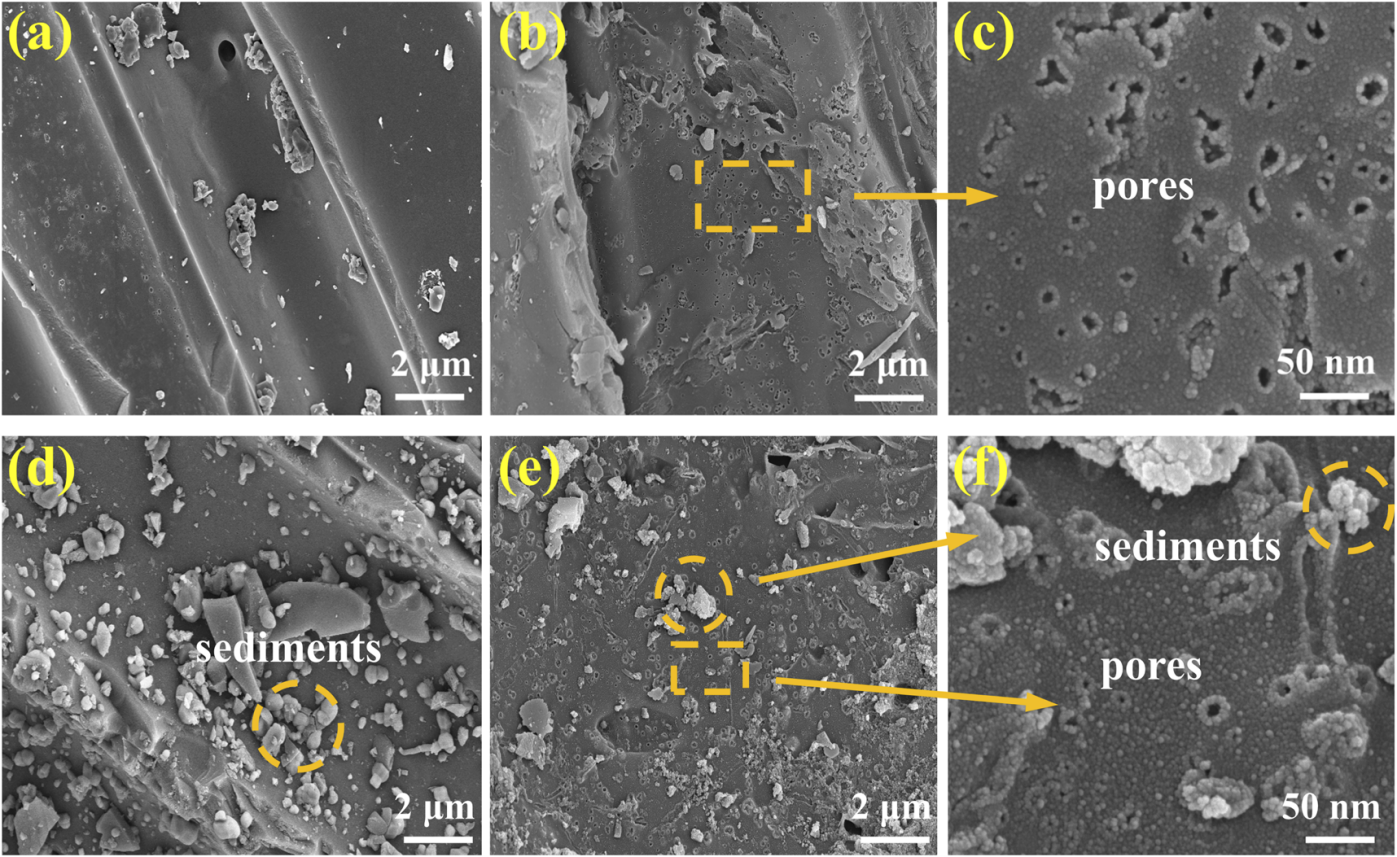
SEM images of the BC (a), OBC (b and c), FBC (d), and FOBC (e and f).

Fig. 1 shows the SEM images of the four adsorbents. The results showed an obvious difference among the surface structures of the four materials. It was clear that BC exhibited an irregular structure with a small number of macropores and a relatively smooth surface (Fig. 1a). On the other hand, OBC exhibited a rough surface structure with surface cracks and numerous mesopores (Fig. 1b and c). Additionally, FBC showed massive sediments on its smooth surface (Fig. 1d). However, FOBC exhibited a rough surface structure with abundant pores and numerous needle-shaped sediment clusters (Fig. 1e and f). Similar structures have been reported in previous related studies.^34,35^ The results from EDS analysis are presented in Fig. S1 and Table 1. The EDS images of BC and OBC showed the absence of elemental Fe. However, both the FBC and FOBC exhibited three extra peaks at 0.71, 6.41, and 7.06 keV, which corresponded to elemental Fe.^36^ According to the EDS results, the contents of Fe and O elements in FOBC were the highest among various materials (Table 1), indicating that FOBC was loaded with more α-FeOOH than FBC, which was due to the reason that Fenton oxidation increased the oxygen-containing functional groups on the surface of FOBC. They can serve as a support to scatter and fix α-FeOOH through either O–metal binding or electrostatic forces.^28^

**Table 1:** The physiochemical properties of samples^a^

Samples	Surface atomic relative content (wt%)			*S*_BET_ (m^2^ g^−1^)	*V*_tot_ (cm^3^ g^−1^)	Average pore diameter (nm)
	C	O	Fe			
BC	91.88	8.12	—	2.2	0.004	7.32
OBC	88.57	11.43	—	100.5	0.102	3.39
FBC	80.79	11.52	7.69	17.3	0.014	3.26
FOBC	70.63	16.86	12.51	60.7	0.042	2.98

a *S*_BET_: BET specific surface area; *V*_tot_: total BJH pore volume.

**Fig. 2:**
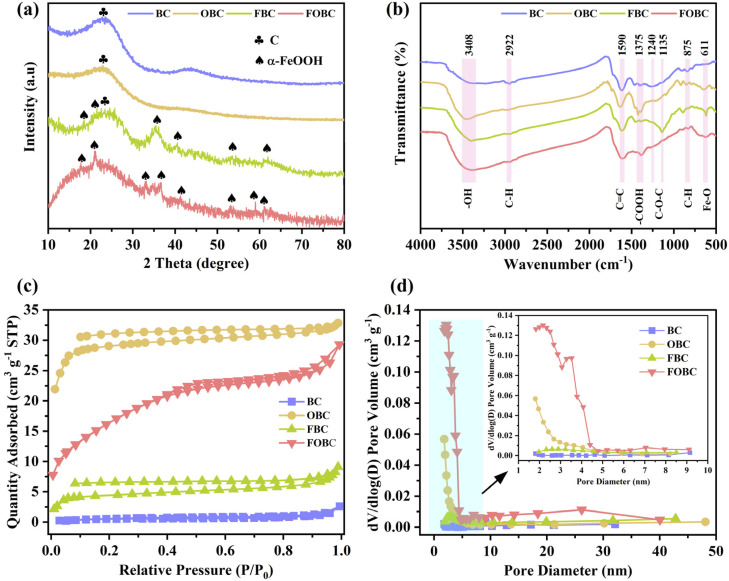
(a) XRD patterns, (b) FTIR spectra, (c) N_2_ adsorption–desorption curves, and (d) BJH pore dimension distributions of the samples.

Fig. 2a shows the XRD patterns of the four adsorbents. The patterns of BC and FBC showed an obvious peak at the 2*θ* value of about 23°, which corresponded to the (002) typical crystal plane of biochar fibers or carbon.^37^ In contrast, the XRD patterns of OBC and FOBC showed comparatively weakened peaks, suggesting the destruction of initial crystallite structure of the biochar surface through Fenton oxidation.^22^ Nevertheless, obvious intense peaks of FBC and FOBC at about 17.81, 21.19, 33.22, 35.37, 36.62, 41.18, 53.15, 59.00, and 61.59° corresponded to the (020), (110), (130), (111), (140), (221), (151), and (002) planes of α-FeOOH (PDF#81-0462), respectively.^35,38^ These findings proved the effective loading of α-FeOOH on biochar substrate.

The obtained FTIR spectra (Fig. 2b) revealed distinct variations in surface group composition of four adsorbents. The BC exhibited a broad band around 3408 cm^−1^, representing the oscillation of structural –OH group.^39^ In addition, characteristic absorption bands emerged at 2922 and 815 cm^−1^ from the vibrational modes of C–H bonds in the aromatic structures, while the signal at 1590 cm^−1^ reflected the stretching of conjugated C=C bond within the aromatic system.^11,40^ The spectral features at 1375 and 1240 cm^−1^ corresponded to the stretching vibrations of structural –COOH and the oscillations of C–O–C, respectively.^39,41^ However, the spectra of OBC and FOBC showed the shift or weaking of peaks at 2922, 1590,1240, and 815 cm^−1^, whereas peaks related to –OH and –COOH became stronger and sharper than those observed in BC. The results suggested the oxidation of C–O–C and C–H bonds within the aromatic rings of biochar's surface through HO˙ radicals, resulting in the formation of –OH and –COOH groups.^22^ Similarly, C=C, which forms the graphitized structure of biochar, was also subjected to oxidation through HO˙, and generated C–OH/–COOH groups, promoting the formation of pores on the surfaces of OBC and FOBC.^10^ Furthermore, compared to OBC, FOBC showed an obvious weaking of peaks related to –OH and –COOH, which was due to the complexation of –OH and –COOH with α-FeOOH.^18,28^ Additionally, FBC exhibited a new and intense peak at 1107 cm^−1^, indicating the inverse stretching vibration of C–O–C bond, which was caused by the binding of α-FeOOH with its O atom.^42^ Notably, a new peak emerged at 611 cm^−1^ in the FTIR spectrum of each of FBC and FOBC, which corresponded to the bending oscillation mode of the Fe–O functional group of α-FeOOH.^30,31^

The N_2_ adsorption–desorption isotherms of the prepared samples are shown in Fig. 2c. The original biochar (BC) exhibited a Type-III isotherm pattern, demonstrating its non-porous nature.^35^ On the other hand, the adsorption curves of the other modified biochar were not overlapped with the desorption curves, indicating pronounced hysteresis loops that were characteristic of Type-IV isotherms. This result indicated the formation of a mesoporous structure after the modification of BC.^24^ In addition, data involving specific surface area and pore volume are summarized in Table 1. Obviously, OBC showed the highest BET specific surface area (100.5 m^2^ g^−1^) and total BJH pore volume (0.102 cm^3^ g^−1^), which were due to the modification of BC through Fenton oxidation, resulting in the expansion of surface cracks and the generation of mesopores. However, after loading α-FeOOH, the FOBC displayed decreases in specific surface area (60.7 m^2^ g^−1^) and total volume (0.042 cm^3^ g^−1^). This might be due to the reason that deposited α-FeOOH occupied some of the surface pores, resulting in decreased surface area and pore volume. Furthermore, compared with BC, the FBC showed a slight increase in specific surface area and total volume, indicating that single modification through loading α-FeOOH could not create sufficient pore structures because of the agglomeration of α-FeOOH.^28^ However, the FOBC still exhibited the enhanced surface characteristics with higher specific surface area and larger pore volume compared to both BC and FBC. Moreover, BJH pore size distributions of the samples were analyzed, as shown in Fig. 2d. The result showed significant structural refinement, with average pore diameters decreasing from 7.32 nm in BC to 2.98 nm in FOBC (Table 1). Therefore, the dual-modification process increased the total pore volume and specific surface area of the biochar, while also decreasing its average pore dimension, thus leading to the availability of more sites for the adsorption of Cu(ii).

### 3.2 Adsorption performance of different adsorbents

Adsorption capacities and efficiencies of four adsorbents for Cu(ii) under the same experimental conditions were investigated, and the corresponding results are shown in Fig. S2. Among the four adsorbents, BC exhibited a poor adsorption performance, with adsorption capacity of 36.7 mg g^−1^ and adsorption efficiency of 26.2%. In contrast, the adsorption capacities and efficiencies of OBC and FBC reached 60.6 mg g^−1^, 43.3% and 76.4 mg g^−1^, 54.6%, respectively. These results suggested that modification through Fenton oxidation or loading α-FeOOH onto the original biochar enhanced the adsorption performance of adsorbent for Cu(ii). However, the adsorption performance of FBC for Cu(ii) was better than that of OBC, which was due to the reason that α-FeOOH had higher affinity for Cu(ii) than oxygen-containing functional groups. Furthermore, FOBC showed much higher adsorption capacity (128.6 mg g^−1^) and efficiency (91.9%) than those of OBC and FBC, indicating the advantages of dual-modification of biochar. This was due to the fact that the previous Fenton oxidation modification achieved an increase in oxygen-containing functional groups, which subsequently resulted in a higher loading of α-FeOOH on the surface of biochar and led to more efficient performance for Cu(ii) adsorption.^28^ Based upon these results, the subsequent experiments were focused on FOBC only.

### 3.3. Effects of parameters on Cu(ii) adsorption by FOBC

#### 3.3.1. Effect of solution pH

**Fig. 3:**
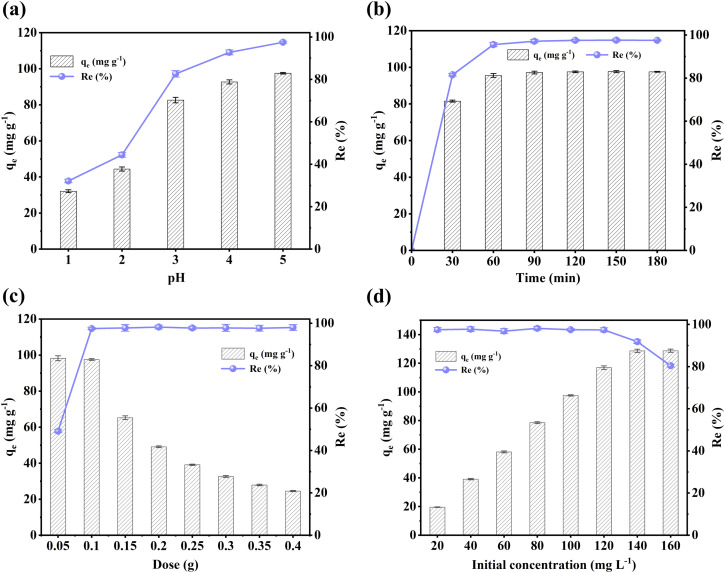
Effects of (a) pH (*C*_0_[Cu(ii)] = 100 mg L^−1^, dose = 0.1 g, *t* = 180 min, *T* = 25 °C), (b) contact time (*C*_0_[Cu(ii)] = 100 mg L^−1^, dose = 0.1 g, *T* = 25 °C, pH = 5), (c) adsorbent dose (*C*_0_[Cu(ii)] = 100 mg L^−1^, *t* = 180 min, *T* = 25 °C, pH = 5), and (d) initial Cu(ii) concentration (dose = 0.1 g, *t* = 180 min, *T* = 25 °C, pH = 5) on the FOBC-based adsorption of Cu(ii).

Solution pH is a prominent factor influencing the adsorption sites, chemical structures of adsorbents, and the forms of HMs.^43^ Cu(ii) is found in a divalent ionic state in solutions, with pH lying within the range of 1–5.^44^ Moreover, the precipitation of Cu(ii) as hydroxides may occur under pH values higher than 5.^45^ Therefore, the present study explored the influence of pH values within the range of 1–5. As shown in Fig. 3a, lower adsorption capacity and efficiency were observed under pH values of less than 3 for FOBC. This could be explained by the protonation of adsorbent surface under lower pH values due to the presence of abundant hydrogen ions, resulting in electrostatic repulsions, which impeded the migration of Cu(ii) to adsorbent sites.^15^ In addition, results showed, with the increase in solution pH, FOBC exhibited increases in the adsorption capacity and efficiency of Cu(ii), reaching values of 97.5 mg g^−1^ and 97.5%, respectively at the pH value of 5. This might be due to the deprotonation of adsorbent surfaces, increasing the negative charge with the increase in pH, which resulted in an increase in the electrostatic attraction between the adsorbent surfaces and Cu(ii).^6^

#### 3.3.2. Effect of contact time

As shown in Fig. 3b, FOBC exhibited the highest adsorption performance for Cu(ii) in the first 30 minutes. In fact, Cu(ii) was quickly adsorbed onto the adsorbent surfaces through mass transfer during the early-stage adsorption, explaining high rates of adsorption for Cu(ii).^46^ However, rates of adsorption for Cu(ii) decreased after 30 minutes, which was attributable to the stepwise saturation of surface adsorption sites. Therefore, it is inferred that Cu(ii) exhibited continuous diffusion into the porous structure through adsorption within pores. It is important to note that, compared to surface adsorption, adsorption within pores is a relatively slow process.^4^ Moreover, FOBC achieved equilibrium adsorption for Cu(ii) at 60 minutes, indicating complete saturation of adsorption sites. These results suggested high rate of adsorption of Cu(ii) by FOBC, which stemmed from the optimized structure of FOBC that endowed it with abundant functional adsorption sites.

#### 3.3.3. Effect of the dose of adsorbent

As shown in Fig. 3c, within the FOBC's dose of 0.05–0.1 g, the almost unchanged adsorption capacity and increased adsorption efficiency for Cu(ii) were observed. The results indicated that the adsorbent remained in saturated adsorption states at lower doses, resulting in unchanged adsorption capacity and increased adsorption efficiency with the increased dose of the adsorbent.^15^ However, the adsorption efficiency of Cu(ii) by FOBC remained unchanged at doses exceeding 0.1 g, with a decreasing trend in adsorption capacity. This might be due to the occurrence of adsorption equilibrium, resulting in a constant concentration of Cu(ii) in solutions, even at higher doses of the adsorbent. There were unoccupied adsorption sites on the surface of adsorbent, reducing the amount of adsorbed Cu(ii) per unit surface area, which resulted in a decrease in the adsorption capacity of Cu(ii).^43,47^

#### 3.3.4. Effect of the initial concentration of Cu(ii)

Fig. 3d shows the effect of initial concentration of Cu(ii) on the adsorption performance of FOBC. For a fixed dose of 0.1 g, FOBC exhibited a progressive enhancement in the adsorption capacity of Cu(ii) until reaching saturation of adsorption for the concentration of 140 mg L^−1^, a value beyond which the adsorption efficiency of FOBC for Cu(ii) decreased due to the continuous saturation of adsorption sites. Moreover, FOBC maintained an adsorption efficiency of about 98% across an initial Cu(ii) concentration of 20–120 mg L^−1^, thereby demonstrating almost complete adsorption of Cu(ii) within these initial concentrations. These findings indicated substantially high adsorption capacity of FOBC, which is mainly attributed to the availability of its abundant adsorption sites to Cu(ii).

### 3.4. Adsorption studies of FOBC

#### 3.4.1. Adsorption kinetics

**Fig. 4:**
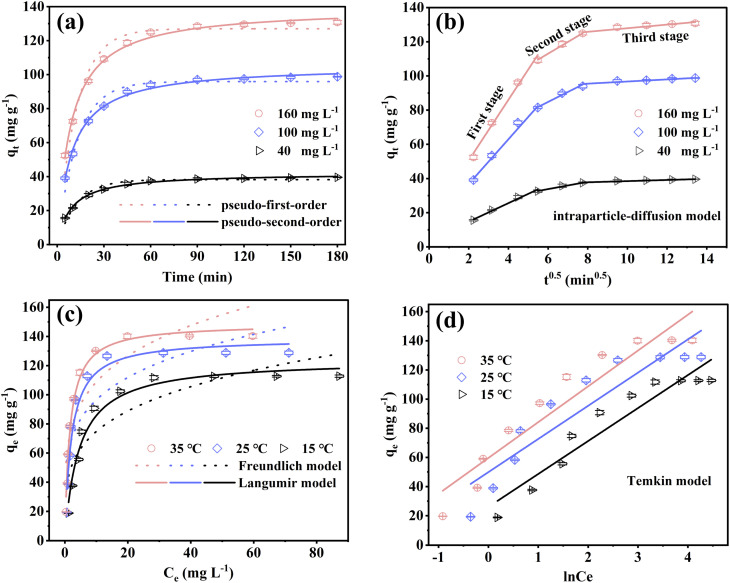
Kinetics (a and b) and isotherm (c and d) of the FOBC-based Cu(ii) adsorption. Conditions: For (a and b), dose = 0.1 g, *t* = 0–180 min, *T* = 25 °C, pH = 5, *C*_0_[Cu(ii)] = 40, 100, and 160 mg L^−1^. For (c and d), *C*_0_[Cu(ii)] = 20–200 mg L^−1^, dose = 0.1 g, *t* = 180 min, pH = 5, *T* = 15, 25, and 35 °C.

Fig. 4a shows the kinetic behavior of Cu(ii) adsorption by FOBC under different initial concentrations. Based on the parameters of the pseudo-first-order and pseudo-second-order kinetic models (Table S1), higher correlation coefficient (*R*^2^) was observed for the pseudo-second-order model (0.994–0.997) than that for the pseudo-first-order model (0.954–0.966). Moreover, the calculated adsorption capacities for Cu(ii) (*q*_e,cal_) derived from the pseudo-second-order model showed a closer agreement with experimental values (*q*_e,exp_), suggesting its appropriateness for describing the adsorption process of Cu(ii).^6,48^ These results also indicated that the adsorption of Cu(ii) using FOBC occurred through chemisorption, which was in agreement with the findings revealed in previous related studies.^7,27,36^

The rate-limiting step in the adsorption of Cu(ii) using FOBC was further explored through the intraparticle-diffusion model (Fig. 4b). The results showed that both curves had three linear parts at each initial concentration of Cu(ii). The finding suggested that the adsorption process of Cu(ii) by FOBC occurred through three distinct stages, including surface film diffusion (first stage), intraparticle-diffusion (second stage), and adsorption equilibrium (third stage).^16,49^ Furthermore, the surface film diffusion rate constants (*k*_pI_) were substantially higher than those of the intraparticle-diffusion (*k*_pII_) at different initial concentrations of Cu(ii) (Table S1), indicating more easier adsorption of Cu(ii) through the boundary layer on the surface than diffusion into the micropores.^24^ The rate constant (*k*_pIII_) values were almost equal to zero, implying that the adsorption of Cu(ii) had reached equilibrium during the third stage. Moreover, the values of *k*_pI_ and *k*_pII_ increased at higher initial concentrations of Cu(ii), which was potentially due to the intensified mass transfer dynamics that were mediated by the concentration of Cu(ii).^27^ The higher initial concentration enhanced the rate of migration of Cu(ii) to the unoccupied surface sites and pores of FOBC. In addition, all fitting lines produced some value for the intercept, implying that the adsorption of Cu(ii) using FOBC was governed by mass transfer and intraparticle diffusion.^50^

#### 3.4.2. Adsorption isotherms

The temperature-dependent characteristics of Cu(ii)'s adsorption process by FOBC were investigated by employing Langmuir and Freundlich's isotherm models, as shown in Fig. 4c. The results from non-linear regression analyses revealed clearly different values for correlation coefficient (*R*^2^) between the Langmuir (*R*^2^ = 0.953–0.981) and Freundlich (*R*^2^ = 0.751–0.808) models under all tested temperatures (Table S2). Therefore, the Langmuir model curves fitted the experimental data more accurately due to higher *R*^2^ values, indicating that Langmuir model was better suited to describe the adsorption of Cu(ii) by FOBC.^4^ This finding implied that monolayer chemisorption was the main mechanism underlying the adsorption of Cu(ii) by FOBC.^51^ More specifically, Cu(ii) might tend to adhere to uniformly active sites by forming chemical bonds.^36^ The Langmuir isotherm model highlighted an increase in the maximum adsorption capacity (*q*_m_) of Cu(ii) with the increase in temperature from 15 to 35 °C. Based upon these results, it can be inferred that increased reaction temperatures are conducive to the adsorption of Cu(ii) by FOBC.

The Temkin isotherm model and its related parameters are presented in Fig. 4d and Table S2, respectively. The results showed an increase in the value of binding constant (*K*_T_) with the increase in temperature from 15 to 35 °C, indicating comparatively stronger affinity between the FOBC active sites and Cu(ii) under higher temperatures.^27^ The calculated values for adsorption heat (B) also increased with the increase in temperature, demonstrating that the adsorption of Cu(ii) by FOBC was endothermic in nature.^52^ In addition, the Langmuir model-based maximum amount of adsorbed Cu(ii) at 35 °C was 148.8 mg g^−1^, which surpassed most of the values reported in contemporary studies (Table 2).

**Table 2:** The maximum adsorption capacity of different adsorbents for Cu(ii)

Adsorbent	pH	Temperature	*q*_m_ (mg g^−1^)	Reference
Bamboo charcoal-layered double hydroxides	5	25 °C	85.47	6
CaFe-layered double hydroxide corn straw biochar	6	45 °C	39.35	16
Iron modified biochar derived from rice husk	5	25 °C	29.78	8
Biochar embedded with hydrated ferric oxide nanoparticles	5	25 °C	33.25	25
KOH-modified bamboo charcoal loaded with α-FeOOH	5	35 °C	117.4	53
Microwave biochar derived from HP pretreated feedstock	5	25 °C	533.34	21
FOBC	5	35 °C	148.8	This work

#### 3.4.3. Adsorption thermodynamics

The thermodynamic characteristics for the adsorption of Cu(ii) by FOBC are presented in Fig. S3 and Table S3. Negative values for Gibbs free energy (Δ*G*^0^) were observed under all tested temperatures. However, the absolute values of Δ*G*^0^ increased with the increase in temperature, indicating spontaneous and thermodynamically favorable characteristics for the adsorption of Cu(ii) by FOBC.^10^ Moreover, the calculated values for the change in enthalpy (Δ*H*^0^) were positive, demonstrating an endothermic characteristic of the adsorption of Cu(ii) by FOBC, which was in agreement with the results obtained from the Temkin model.^50^ Values for the change in entropy (Δ*S*^0^) were also positive, suggesting the occurrence of preferential surface adsorption over pore diffusion during the adsorption of Cu(ii) and resulting in an increase in the disorder of solid–liquid interface.^46,52^

### 3.5. Adsorption mechanism

#### 3.5.1. FTIR analysis

**Fig. 5:**
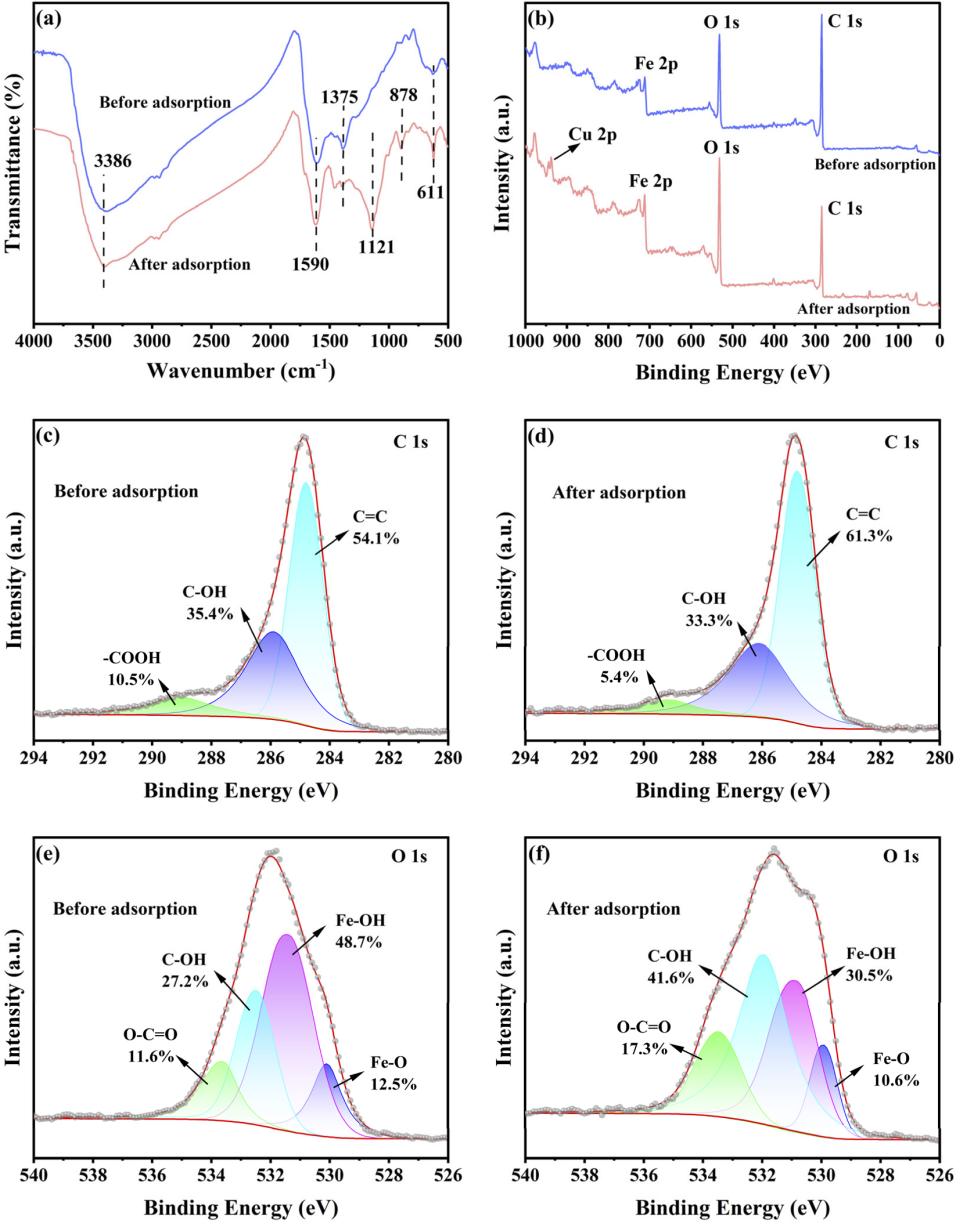
Spectra of the FOBC before and after Cu(ii) adsorption. FTIR (a); full scan XPS (b); XPS C 1s before (c) and after (d) Cu(ii) adsorption; XPS O 1s before (e) and after (f) Cu(ii) adsorption.

The functional groups responsible for the adsorption of Cu(ii) were identified by employing FTIR analysis. Fig. 5a shows the FTIR spectra of FOBC both prior to and following the adsorption of Cu(ii). Peaks at 3386 and 1590 cm^−1^ (–OH and C=C, respectively) exhibited shifts, implying that the C=C and –OH groups on the surface of FOBC were involved in the adsorption of Cu(ii). This could be due to the interaction of non-bonding electron pairs of oxygen atoms in the –OH group with Cu(ii) through coordination reactions, forming surface complexes (such as –O–Cu) or substitution of H through ion exchange.^16,54^ The presence of a delocalized electron related to C=C group might promote the combination of C=C and Cu(ii) through cation-π coordination mechanism.^55,56^ On the other hand, peaks at 1375 cm^−1^ (–COOH group) and 611 cm^−1^ (Fe–O) became weaker or slightly shifted, indicating the involvement of –COOH and α-FeOOH in the adsorption of Cu(ii). In addition, new peaks were observed at 1121 and 878 cm^−1^ following the adsorption of Cu(ii). These peaks might be attributable to the formation of surface complexes, including C–O–Cu and Fe–O–Cu, through coordination reactions and ion exchange, respectively.^41^

#### 3.5.2. XPS analysis

To comprehensively explore the adsorption mechanism of Cu(ii) onto FOBC, the high-definition XPS spectra were analyzed both before and after the adsorption of Cu(ii) (Fig. 5b–f). The results showed that FOBC mainly contained C, O, and Fe. In addition, signals for Cu element were detected at about 935.1 eV, showing an obvious peak after the adsorption process, and confirming the successful adsorption of Cu(ii) (Fig. 5b). This was in agreement with the EDS element mappings shown in Fig. S4.

In addition, the deconvolution of C 1s XPS spectrum (Fig. 5c and d) manifested three distinct peaks at 284.8, 286.1, and 289.1 eV, which corresponded to C=C, C–OH, and –COOH bonds, respectively.^23,57^ Following the adsorption of Cu(ii), the relative content of C=C increased from 54.1% to 61.3%. In contrast, the relative contents of C–OH and –COOH decreased from 35.4% to 33.3% and from 10.5% to 5.4%, respectively. These findings demonstrated that, on the surface of FOBC, the functional groups of –OH and –COOH exhibited a stronger affinity for Cu(ii) than the C=C group.^16^ It should be noted that the relative content of –COOH decreased by 48.6%. This decreasing rate was, in fact, substantially higher than that of the relative C–OH content (5.9%). Therefore, –COOH possessed a higher affinity for Cu(ii) than –OH. This might be due to the lone-pair electrons of –COOH oxygen atoms, which resulted in stronger electron-donating power than that of –OH.^58^ The cation-π coordination interaction between Cu(ii) and C=C was substantially weaker than that between oxygen-containing functional groups and Cu(ii).^56^ Therefore, the contributions of various functional groups to the adsorption of Cu(ii) by FOBC followed the descending order of: –COOH > –OH > C=C.

As presented in Fig. 5e and f, the O 1s XPS spectrum was deconvoluted into four peaks at binding energies of about 533.5, 532.3, 531.1 and 529.7 eV, which were attributed to O–C=O, C–OH, Fe–OH, and Fe–O bond, respectively.^34,35,39,49^ In addition, the O 1s spectrum exhibited a noticeable change following the adsorption of Cu(ii). The relative contents of C–OH and O–C=O increased from 27.2% and 11.6% to 41.6% and 17.3%, respectively. In contrast, the relative contents of Fe–OH and Fe–O decreased from 48.7% and 12.5% to 30.5% and 10.6%, respectively. These results suggested that –COOH and –OH were not the main factors contributing to the adsorption of Cu(ii). In contrast, α-FeOOH on FOBC surfaces exhibited a pivotal role in the adsorption of Cu(ii). These findings can be explicated by the fact that α-FeOOH was more likely to undergo a coordination reaction with Cu(ii) because of its higher affinity for cations when compared with –COOH and –OH.^49,59^ Moreover, the proportion of Fe–OH/Fe–O decreased from 3.90 to 2.88 following the adsorption of Cu(ii), demonstrating the robust coordination between α-FeOOH-derived Fe–OH and Cu(ii) through different pathways. These pathways might include cation exchange involving the substitution of H and the exchange or transfer of electron during the adsorption process, which contributed to the formation of a more stable Fe–O–Cu complex.^41^

#### 3.5.3. DFT calculations for adsorption mechanism

**Fig. 6:**
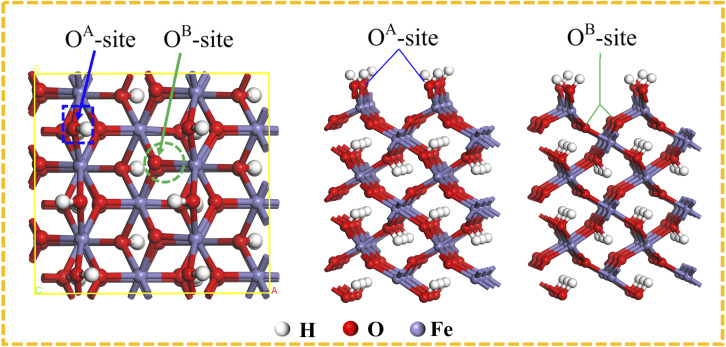
O^A^-site and O^B^-site on the α-FeOOH surface.

Given the most important role of α-FeOOH in the adsorption of Cu(ii), the DFT calculations were conducted to comprehensively explore the micro-mechanism underlying the α-FeOOH-based adsorption of Cu(ii) from an electronic perspective. Previous studies have indicated two types of oxygen atoms on the (110) α-FeOOH surface, representing the Fe–O–H and Fe–O–Fe structures and forming two main cation adsorption sites of O^A^-site and O^B^-site, respectively.^32^Fig. 6 shows the two adsorption sites on the surface of α-FeOOH. The adsorption configurations of Cu(ii) prior to and following the optimization process are shown in Fig. S5. The results showed a lower *E*_ads_ value for the adsorbed Cu(ii) on the O^A^-site (−7.36 eV) than that on the O^B^-site (−6.47 eV) following the calculation of the adsorption configuration. Therefore, the O^A^-site of Fe–O–H structure was the most stable configuration for α-FeOOH-based adsorption of Cu(ii) due to a more negative *E*_ads_ value.^60–62^ Moreover, the negative *E*_ads_ values indicated that the α-FeOOH-based adsorption of Cu(ii) was a spontaneous process.^63^

**Fig. 7:**
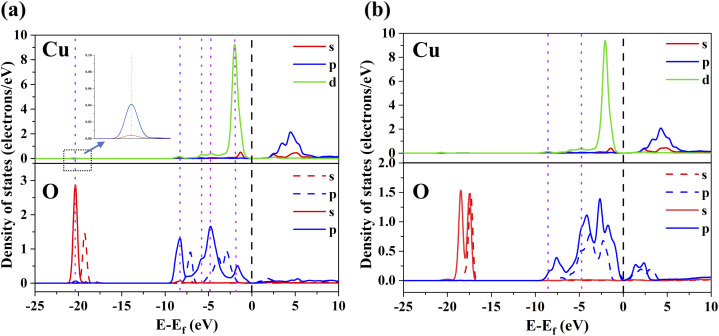
DOS of Cu, O atom before (dashed line) and after (solid line) Cu(ii) adsorption on the O^A^-site (a) and O^B^-site (b). The black and purple vertical dotted lines indicate the Fermi level and orbital overlaps, respectively.

To comprehensively analyze the roles of atomic orbitals in the adsorption process, the density of states (DOS) of O and Cu were computed based on the changes in electronic structures (Fig. 7). According to Fig. 7a, the atomic orbitals of O in the left part of the Fermi energy level carried out a transition to low-energy state after the adsorption of Cu(ii), increasing the corresponding densities of s and p electrons. This finding demonstrated the occurrence of electron transfer through the adsorption of Cu(ii) on O^A^-site.^33,64^ Moreover, the s, p, and d orbitals of Cu(ii) overlapped well with the s and p orbitals of O atom of O^A^-site, further indicating the strong chemical interaction between Cu(ii) and O^A^-site through charge transfer and orbital hybridization.^60,62^ As illustrated in Fig. 7b, only the p-orbital of O atom of O^B^-site slightly overlapped with the d-orbital of Cu(ii). Therefore, the orbital overlap of Cu(ii) and O^B^-site was obviously lower than that of Cu(ii) and O^A^-site. This might be due to the smooth substitution of Fe–O–H-derived H, which resulted in the generation of lone-pair electrons of O^A^-site that promoted deeper orbital hybridization between Cu(ii) and O^A^-site than that between Cu(ii) and O^B^-site.^64,65^ This also explains the stronger adsorption stability and affinity of the O^A^-site for Cu(ii) than the O^B^-site.

**Fig. 8:**
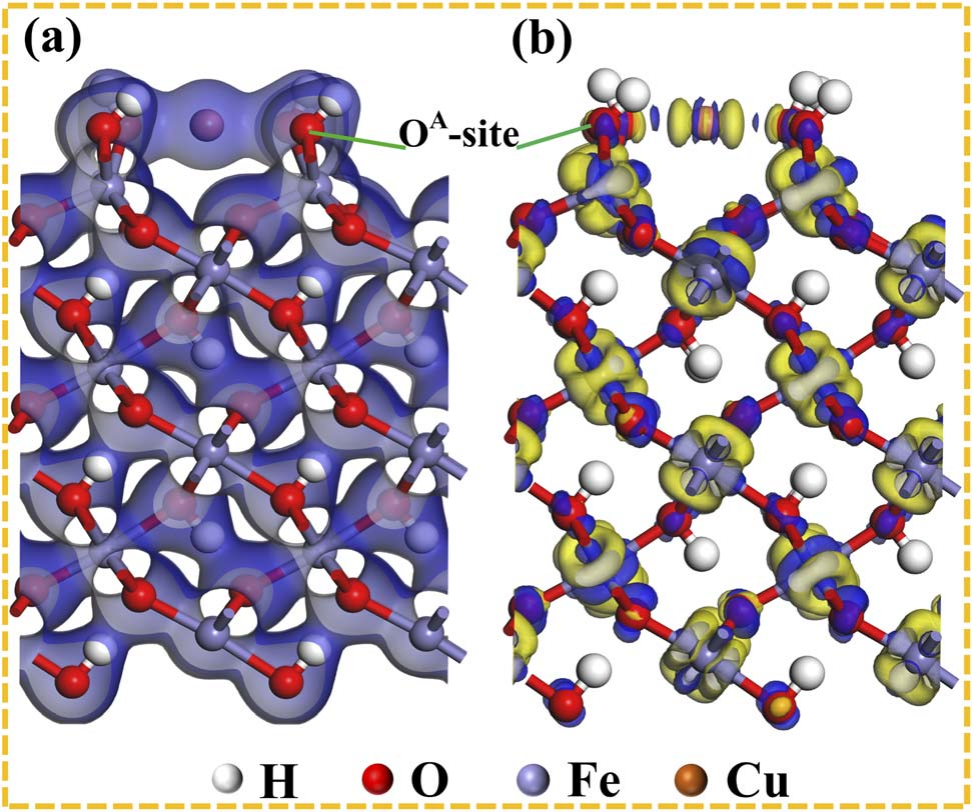
Electron density (a) and differential charge (b) maps of Cu(ii) adsorption configurations on the O^A^-site.

To investigate the electron transfer or exchange behaviors during the adsorption of Cu(ii), the electron density and differential charge were also analyzed. Fig. 8a shows the electron density map of Cu(ii)'s adsorption on O^A^-site. The electronic cloud of adsorbed Cu(ii) almost symmetrically overlapped with the electronic cloud of the two O^A^-sites. Indeed, the symmetrical extension of the electron cloud's overlapping can well explain the electron exchange during the Cu(ii)'s adsorption onto O^A^-sites.^66^ The differential charge map of the adsorbed Cu(ii) on O^A^-site is shown in Fig. 8b. The yellow and blue areas indicated the acquisition and donation of electrons, respectively.^58^ The adsorbed Cu(ii) (yellow color) suggested the demand for electrons. In contrast, the O atoms of O^A^-sites are shown in blue (in Fig. 8b), suggesting that the adsorption process of Cu(ii) was driven by the donation of electrons from O^A^-sites.^66^ In addition, the differential charge map revealed symmetrical electron transfer from O^A^-sites to Cu(ii) (Fig. 8b). These findings demonstrated the possibility of adsorbing Cu(ii) on the O^A^-sites of α-FeOOH through electron exchange or transfer to form a stable bidentate complex, representing the main FOBC-based Cu(ii) adsorption process, according to the following chemical reaction: 2 ≡Fe–OH + Cu(ii) → ≡Fe–O–Cu–O–Fe≡ + 2H^+^.

These findings revealed that the FOBC-based adsorption of Cu(ii) primarily occurred through ion exchange and coordination interactions. The contributions of FOBC's functional groups lied in the descending order of: α-FeOOH ≫ –COOH > –OH > C=C. In addition, the structure of Fe–O–H of α-FeOOH played an overriding role during the adsorption of Cu(ii).

### 3.6. Influences of co-existing ions

**Fig. 9:**
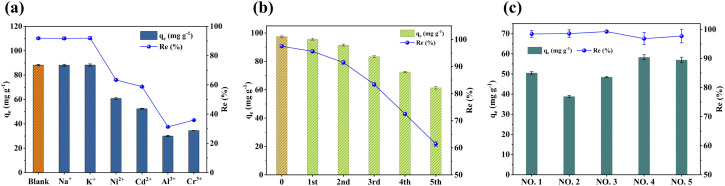
Influences of co-existing ions on Cu(ii) adsorption (a), the performance of FOBC for treating actual Cu(ii)-containing wastewater (b), and the reusability of FOBC for Cu(ii) after adsorption–desorption (c). Conditions: For (a), dose = 0.1 g, *t* = 180 min, *T* = 25 °C, pH = 4, C_0_[Cu(ii)] = 1.5 mmol L^−1^, initial Cu(ii) and co-existing ions (Na^+^, K^+^, Ni^2+^, Cd^2+^, Al^3+^, and Cr^3+^) at a molar ratio of 1 : 1. For (b), dose = 0.1 g, *t* = 180 min, *T* = 25 °C, pH = 5, C_0_[Cu(ii)] = 100 mg L^−1^. For (c), dose = 0.1 g, *t* = 180 min, the quality parameters of wastewater are reported in Table S4.

In general, some ions, such as heavy metals and common salt ions, can co-exist in actual Cu(ii)-containing wastewater. Fig. 9a shows the competitive effects of different ions on the adsorption of Cu(ii) by FOBC. According to the results, co-existing monovalent ions (Na^+^ and K^+^) almost did not exert any effect on the adsorption of Cu(ii). The finding might be due to the low charge densities of monovalent ions, making orbital hybridization with the O atom of adsorption sites difficult and resulting in stronger interactions with H_2_O.^64^ On the other hand, divalent ions (Ni^2+^ and Cd^2+^) exerted a stronger influence on the adsorption of Cu(ii) by FOBC, implying a pronounced competitive effect for Cu(ii). Additionally, the influences of trivalent ions (Al^3+^ and Cr^3+^) on the adsorption of Cu(ii) by FOBC were substantially stronger than those of the divalent ions, indicating a relatively high binding affinity between the trivalent ions and the adsorption sites. The phenomenon might be due to the high positive charge of trivalent ions, enhancing the orbital attraction to adsorption sites and reducing the kinetic barrier of the adsorption reaction.^64,67^ Nevertheless, it should be emphasized that FOBC maintained an adsorption efficiency of more than 30% for Cu(ii) under the initial simultaneous presence of Cu(ii) and Al^3+^/Cr^3+^ at a molar ratio of 1 : 1.

### 3.7. Potential of FOBC

Recyclability, stability, and feasibility are prominent indicators to determine the application potential of adsorbents.^68^ In the present work, five consecutive adsorption–desorption cycles were conducted to analyze the reusability of FOBC (Fig. 9b). The results revealed a gradual decline in the adsorption capacity for Cu(ii) with successive regeneration cycles, which was potentially attributed to the partial depletion of adsorption sites following the regeneration process. However, the adsorption efficiency and capacity of FOBC for Cu(ii) at the fifth cycle were 61.4% and 61.4 mg g^−1^, respectively, corresponding to 63% of the first adsorption performance, indicating good reusability of the FOBC. Additionally, SEM images showed a lack of significant effects on the FOBC surface morphology before and following the Cu(ii) adsorption (Fig. S4a and d), demonstrating good stability of the structure of FOBC.^7^

Furthermore, Fig. 9c shows removal performance of Cu(ii) by FOBC when used to treat Cu(ii)-containing industry wastewater. The results showed that the adsorption efficiency for Cu(ii) in all five samples was close to 100%, highlighting the feasibility of FOBC to be used as an efficient adsorbent to remove Cu(ii) from industrial wastewater. Consequently, these findings collectively demonstrate FOBC's excellent potential, making it an appropriate eco-friendly material for the elimination of Cu(ii)from wastewater.

## 4. Conclusions

In the present work, FOBC was successfully synthesized using dual-modification strategy. According to characterization data, the Fenton oxidation resulted in the expansion of specific surface area and increase in oxygen-containing groups, facilitating the subsequent loading of α-FeOOH on the surface of FOBC. Furthermore, the FOBC-based adsorption of Cu(ii) was found to be consistent with the pseudo-second-order kinetics and Langmuir models. Based upon Langmuir's model, the obtained maximum adsorption capacity (*q*_m_) of Cu(ii) by FOBC was 148.8 mg g^−1^ at 35 °C. The characterization analyses revealed that the contribution of various surface groups to the Cu(ii) adsorption process followed the order of α-FeOOH ≫ –COOH > –OH > C=C. However, the quantification of adsorption capacities of various functional groups on Cu(ii) at the micro level is still missing from literature, requiring follow-up studies. The present paper highlights a novel method for developing dual-modified adsorbents from biochar, and provides a potential eco-friendly pathway for treating Cu(ii)-containing wastewater.

## Conflicts of interest

There are no conflicts to declare.

## Supplementary Material

RA-015-D5RA05887B-s001

## Data Availability

The data are available from the corresponding author on reasonable request. Supplementary information: EDS data, SEM images, DFT calculation results, adsorption models and parameters. See DOI: https://doi.org/10.1039/d5ra05887b.
