# Supplementary material for: Dual-modification of biochar via Fenton oxidation and in situ α-FeOOH synthesis for enhanced Cu(ii) removal: experimental investigation and theoretical calculation analysis
Source: RSC Adv. 2025 Nov 18;15(53):44961–73. doi: 10.1039/d5ra05887b (PMC12625812; doi:10.1039/d5ra05887b)
Supplement: RA-015-D5RA05887B-s001 [file RA-015-D5RA05887B-s001.pdf]

Supplementary Information (SI) for RSC Advances.
This journal is © The Royal Society of Chemistry 2025

Dual-modification of biochar via Fenton oxidation and in-situ α-FeOOH synthesis for enhanced Cu(II) removal: Experimental investigation and theoretical calculation analysis

Wei Yang [a, b], Ziguo Liu [a, b, *], Qingkun Liu [c], Qingbin Sun [a], Han Zheng [a, b], Jiancheng Hu [a, b]

[a] Huangshi HBPU Environmental Protection and Energy Saving Industry Technology Research Institute Co., Ltd, Huangshi 435003, China.

[b] School of Environmental Science and Engineering, Hubei Polytechnic University, Huangshi 435003, China.

[c] School of Materials Science and Engineering, Henan Polytechnic University, Jiaozuo 454003, China.

* Corresponding author. E-mail address: liuziguo@hbpu.edu.cn

Text S1

Adsorption kinetics, isotherms, thermodynamic models

1. Kinetics models

To determine the rates and mechanisms of the Cu(II) adsorption process in this study, the pseudo-first-order (Eq. 5), pseudo-second-order (Eq. 6), and intra-particle diffusion (Eq. 7) models were employed, according to the following equations:[1,2]

$$q_t = q_e\left(1 - e^{-k_1 t}\right) \tag{1}$$

$$q_t = \frac{k_2 q_e^2 t}{1 + k_2 q_e t} \tag{2}$$

$$q_t = k_p t^{0.5} + C \tag{3}$$

where k_1 (min^{-1}), k_2 (g mg^{-1} min^{-1}), and k_p (mg g^{-1} min$^{-0.5}$) are the reaction rate constants of each model; C (mg g^{-1}) is constant related to diffusion.

2. Isotherm models

In this study, the Langmuir (Eq. 8), Freundlich (Eq. 9), and Temkin (Eq. 10) isotherm models were used to fit the experimental data and evaluate the Cu(II) adsorption capacity and performance of FOBC, according to the following equations:[3]

$$q_e = \frac{K_L q_m C_e}{1 + K_L C_e} \tag{4}$$

$$q_e = K_F C_e^{1/n} \tag{5}$$

$$q_e = BlnK_T + BlnC_e \tag{6}$$

where q_m (mg g^{-1}) denotes the maximum adsorbed amount of Cu(II); K_L (L mg^{-1}) represents the Langmuir equilibrium constant; K_F (mg g^{-1}) is the Freundlich constant related to adsorption ability; n denotes the adsorption intensity; B (J mol^{-1}) denotes the Temkin constant related to the adsorption heat; T (K) denotes the absolute temperature; K_T (L g^{-1}) is the equilibrium binding constant.

3. Adsorption thermodynamics

To examine the spontaneity and feasibility of the Cu(II) adsorption by FOBC, three thermodynamic factors, including Gibbs free energy (ΔG^0), enthalpy (ΔH^0), and entropy (ΔS^0), were calculated using the following equations:[4]

$$\Delta G^0 =- RT\ln K_C \tag{7}$$

$$\ln K_C = \frac{\Delta S^0}{R} - \frac{\Delta H^0}{RT} \tag{8}$$

$$K_C = M \times 55.5 \times 1000 \times K_L \tag{9}$$

where R (8.314 J mol^{-1} K^{-1}) is the gas constant; K_C is the coefficient related to Langmuir constant K_L; M (g mol^{-1}) denotes the adsorbate molar mass: 55.5 corresponds to the solvent molar concentration (mol L^{-1}).

References

1. M. A. Al-Ghouti and D. A. Da'ana, *J. Hazard. Mater.*, 2020, **393**, 122383.

2. J. E. S. Pereira, R. L. S. Ferreira, P. F. P. Nascimento, A. J. F. Silva, C. E. A. Padilha and E. L. Barros Neto, *Environ. Technol. Innov.*, 2021, **23**, 101706.

3. Y. Jin, M. Zhang, Z. Jin, G. Wang, R. Li, X. Zhang, X. Liu, J. Qu and H. Wang, *Environ. Res.*, 2021, **196**, 110323.

4. X. Zhou and X. Zhou, *Chem. Eng. Commun.*, 2014, **201**, 1459-1467.

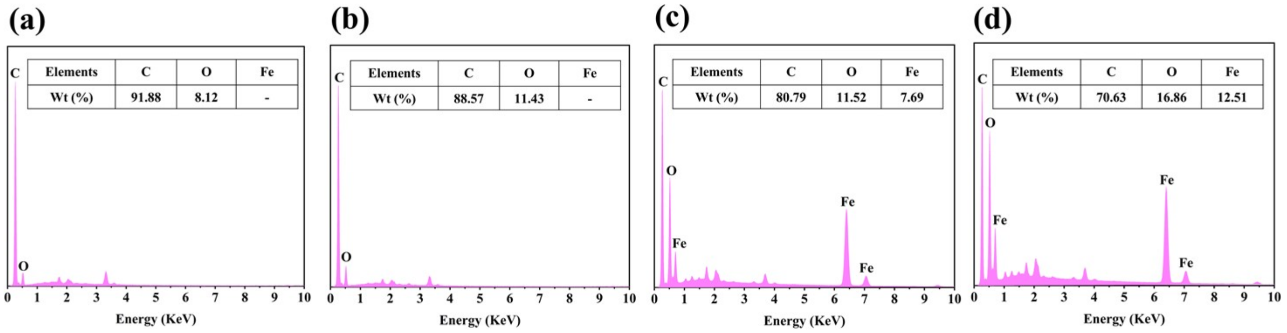

Fig. S1. EDS images of (a) BC, (b) OBC, (c) FBC, and (d) FOBC.

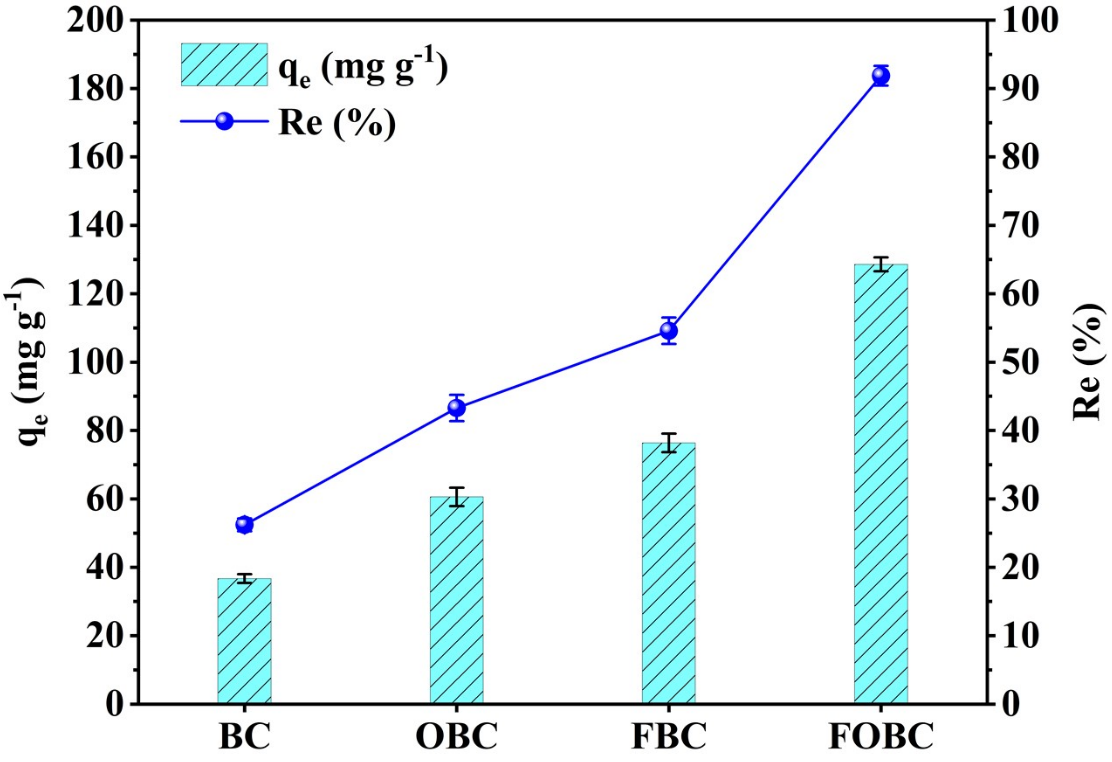

Fig. S2. Comparison of different adsorbents on the adsorption performance for Cu(II). Conditions: C_0[Cu(II)] = 140 mg L^{-1}, adsorbent dose = 0.1 g, t = 180 min, T = 25 °C, pH = 5.

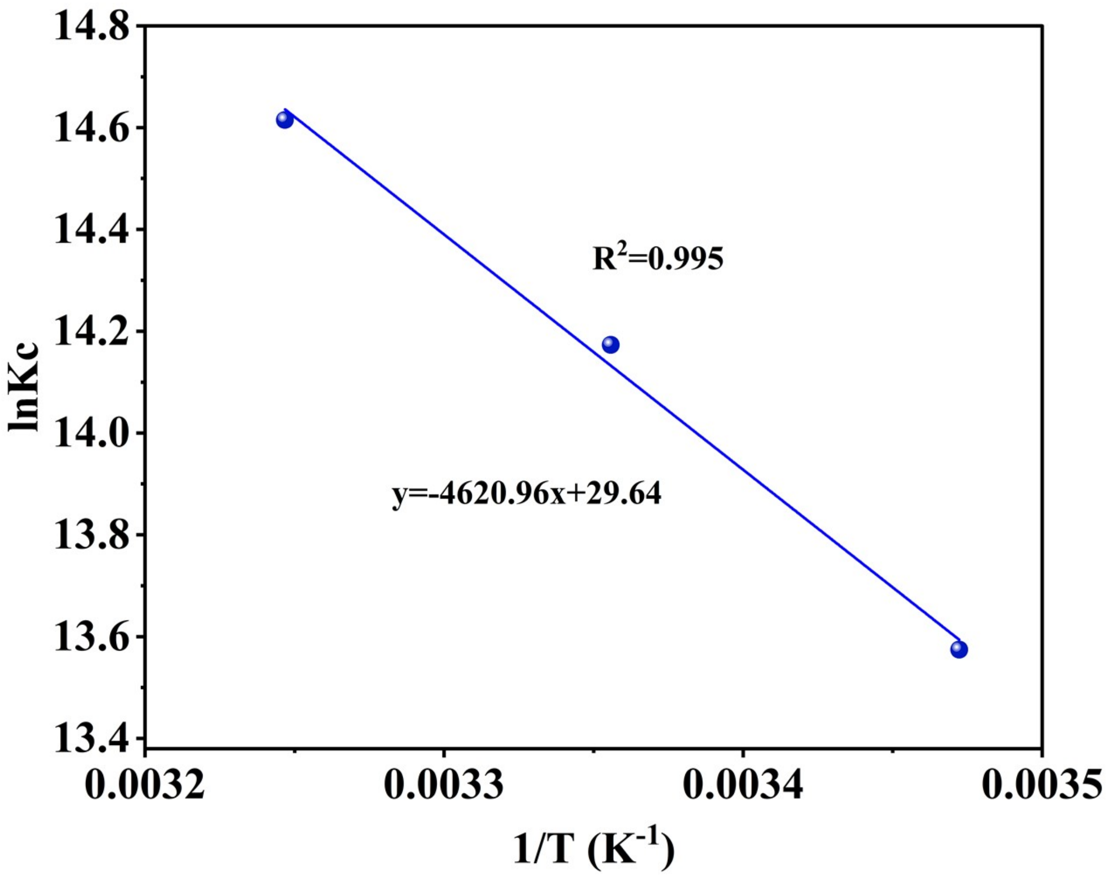

Fig. S3. Thermodynamic analysis of Cu(II) adsorption by FOBC.

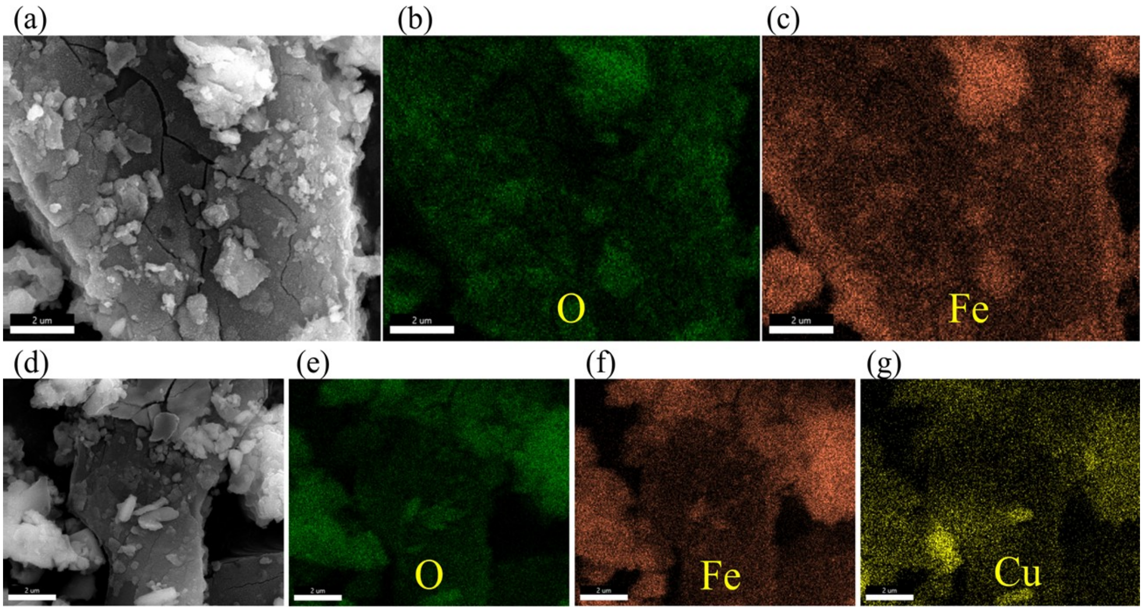

Fig. S4. SEM images of FOBC before (a), and after (d) Cu(II) adsorption; EDS elemental mappings of O (b), Fe (c) before, and O (e), Fe (f), Cu (g) after Cu(II) adsorption.

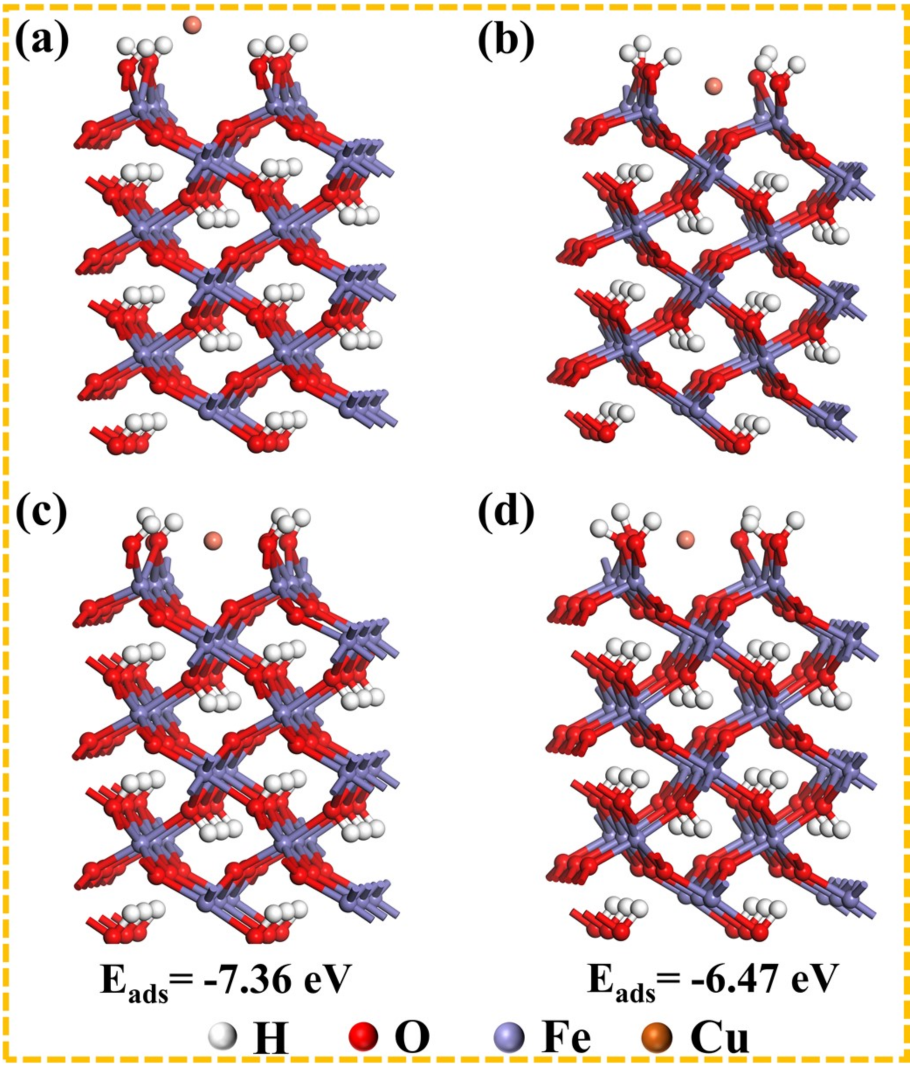

Fig. S5. The initial configuration with the O^A-site (a), O^B-site (b) before Cu(II) adsorption; the optimized configuration and the calculated E_{ads} with the O^A-site (c), O^B-site (d) after Cu(II) adsorption.

Table S1

Kinetics parameters for Cu(II) adsorption at different initial concentration.

Parameters	Initial Cu(II) concentration		
	40 mg L^{-1}	100 mg L^{-1}	160 mg L^{-1}
$q_{e,exp}$ (mg g^{-1})	39.6 ± 0.1	98.8 ± 0.2	130.8 ± 1.1
**Pseudo-first-order**			
$q_{e,cal}$ (mg g^{-1})	40.8	102.5	135.3
k_1 (min^{-1})	0.079	0.078	0.080
R_1^2	0.965	0.966	0.954
**Pseudo-second-order**			
$q_{e,cal}$ (mg g^{-1})	38.2	94.2	124.7
k_2 *10^3 (g mg^{-1} min^{-1})	2.7	1.1	0.8
R_2^2	0.996	0.994	0.997
**Intraparticle-diffusion**			
k_{pI} (mg g^{-1} $min^{-0.5}$)	5.42	13.08	17.72
C_1 (mg g^{-1})	3.94	10.68	15.24
R_{3I}^2	0.989	0.993	0.989
k_{pII} (mg g^{-1} $min^{-0.5}$)	2.13	5.84	6.88
C_2 (mg g^{-1})	21.01	49.76	71.81
R_{3II}^2	0.995	0.981	0.996
k_{pIII} (mg g^{-1} $min^{-0.5}$)	0.33	0.62	1.05
C_3 (mg g^{-1})	35.14	90.55	117.40
R_{3III}^2	0.976	0.949	0.932

Table S2

Isotherm parameters for Cu(II) adsorption at different temperatures.

Parameters	Temperature		
	15 ℃	25 ℃	35 ℃
$q_{e,exp}$ (mg g^{-1})	112.8 ± 0.78	128.8 ± 1.18	140.3 ± 1.50
**Langmuir isotherm**			
$q_{m,cal}$ (mg g^{-1})	124.1	138.7	148.8
K_L (L mg^{-1})	0.223	0.406	0.631
R_1^2	0.973	0.953	0.981
**Freundlich isotherm**			
K_F (mg g^{-1}) (mg L^{-1})^n	42.21	58.98	65.52
n (g mg^{-1} min^{-1})	4.02	4.68	4.55
R_2^2	0.808	0.751	0.799
**Temkin isotherm**			
B (J mol^{-1})	22.60	22.76	24.57
K_T (g L^{-1})	3.16	8.95	11.32
R_3^2	0.904	0.848	0.901

Table S3

Thermodynamic factors for Cu(II) adsorption at different temperatures.

Temperature	Thermodynamic parameters		
	ΔG^0 (kJ mol^{-1})	ΔS^0 (J mol^{-1} K^{-1})	ΔH^0 (kJ mol^{-1})
15 ℃	-32.50	246.43	38.42
25 ℃	-35.12		
35 ℃	-37.43		

Table S4

Water quality parameters of actual Cu(II)-containing wastewater.

Samples	pH	Ions ($mg\ L^{-1}$)									
		Na^+	NH_4^+	**Cu(II)**	Pb(II)	Sn(II)	Ni(II)	Cr(III)	SO_4^{2-}	NO_3^-	Cl^-
NO. 1	3.2	31.5	-	**39.4**	2.1	1.2	1.6	-	40.9	62.1	30.6
NO. 2	3.8	24.6	5.5	**48.8**	1.9	3.8	0.3	0.2	45.8	80.3	27.8
NO. 3	3.6	45.3	7.6	**51.3**	1.4	1.6	1.5	0.6	36.4	107.3	58.5
NO. 4	3.9	36.4	10.3	**60.2**	-	0.7	2.4	-	46.7	48.2	85.3
NO. 5	3.1	28.9	3.2	**58.3**	0.5	-	1.8	0.1	73.6	51.7	35.5

* The actual Cu(II)-containing wastewater was the supernatant of the comprehensive regulating pool collected from a factory in Huangshi, Hubei province, China.
